# Development of a SNP resource and a genetic linkage map for Atlantic cod (*Gadus morhua*)

**DOI:** 10.1186/1471-2164-11-191

**Published:** 2010-03-22

**Authors:** Sophie Hubert, Brent Higgins, Tudor Borza, Sharen Bowman

**Affiliations:** 1 The Atlantic Genome Centre, Halifax, NS, B3H 3Z1, Canada; 2 Department des Sciences, Universite Sainte Anne, Pointe-de-l'Eglise, NS, B0W 1M0, Canada

## Abstract

**Background:**

Atlantic cod (*Gadus morhua*) is a species with increasing economic significance for the aquaculture industry. The genetic improvement of cod will play a critical role in achieving successful large-scale aquaculture. While many microsatellite markers have been developed in cod, the number of single nucleotide polymorphisms (SNPs) is currently limited. Here we report the identification of SNPs from sequence data generated by a large-scale expressed sequence tag (EST) program, focusing on fish originating from Canadian waters.

**Results:**

A total of 97976 ESTs were assembled to generate 13448 contigs. We detected 4753 SNPs that met our selection criteria (depth of coverage ≥ 4 reads; minor allele frequency > 25%). 3072 SNPs were selected for testing. The percentage of successful assays was 75%, with 2291 SNPs amplifying correctly. Of these, 607 (26%) SNPs were monomorphic for all populations tested. In total, 64 (4%) of SNPs are likely to represent duplicated genes or highly similar members of gene families, rather than alternative alleles of the same gene, since they showed a high frequency of heterozygosity. The remaining polymorphic SNPs (1620) were categorised as validated SNPs. The mean minor allele frequency of the validated loci was 0.258 (± 0.141). Of the 1514 contigs from which validated SNPs were selected, 31% have a significant blast hit. For the SNPs predicted to occur in coding regions (141), we determined that 36% (51) are non-synonymous. Many loci (1033 SNPs; 64%) are polymorphic in all populations tested. However a small number of SNPs (184) that are polymorphic in the Western Atlantic were monomorphic in fish tested from three European populations. A preliminary linkage map has been constructed with 23 major linkage groups and 924 mapped SNPs.

**Conclusions:**

These SNPs represent powerful tools to accelerate the genetic improvement of cod aquaculture. They have been used to build a genetic linkage map that can be applied to quantitative trait locus (QTL) discovery. Since these SNPs were generated from ESTs, they are linked to specific genes. Genes that map within QTL intervals can be prioritized for testing to determine whether they contribute to observed phenotypes.

## Background

With many wild Atlantic cod (*Gadus morhua*) stocks declining dramatically over the last few decades [1], aquaculture has become increasingly important as a means of maintaining a market supply for this species. Cod aquaculture is currently being developed in several countries [2], but has not yet reached a sustainable commercial scale [3]. Applying genomics tools in the selection of elite broodstock has the potential to enhance the productivity and value of commercial production for this species [4].

Genetic marker discovery is a necessary first step in the application of genomics to improve broodstock as these markers can be used for the creation of linkage maps and subsequent QTL identification. Marker assisted selection (MAS) can then be employed by selecting broodstock based on genotypes at QTL that are relevant to economically important traits such as rapid growth, disease resistance and the control of early maturation. Currently, a limited collection of genetic markers is available for Atlantic cod, including restriction fragment length polymorphisms (RFLP), microsatellites and single nucleotide polymorphisms (SNPs) [5-9]. Many of the studies using genetic markers to analyse population structure in Atlantic cod have employed microsatellite markers [10-12], and microsatellites have been used extensively in cod aquaculture [13-15]. In total, 352 microsatellites have been published to date for this species, including a large, new collection of expressed sequence tag (EST) derived microsatellites [8]. However, SNPs are the most abundant type of DNA sequence polymorphism, are suitable for high-throughput genotyping, and provide enhanced possibilities for genetic and breeding applications, linkage map development, assessment of genetic variability and marker assisted breeding. As a result, SNP discovery pipelines have been recently developed for many species including fishes [16-22]. To date, a collection of 318 SNPs has been identified for Atlantic cod using 17,056 ESTs generated from a North-East Atlantic cod population, and these SNPs have been tested on several additional Norwegian cod populations [23]. In total, 174 of these SNPs, together with 33 microsatellites, have been used to generate a genetic linkage map for Atlantic cod. This map comprises 25 linkage groups with an overall length of 1225 cM, and represents the first reported linkage map for this species [24].

The Cod Genomics and Broodstock Development Project (CGP) recently produced 158,877 expressed sequence tags (ESTs) [25] using a large number of individuals and a variety of cDNA libraries, including several blood, embryonic and larval normalized cDNA libraries. In the present study, we used this collection of sequences to identify SNPs. Our EST set was designed to provide an excellent resource for SNP marker discovery, since it is generated from several cDNA libraries representing different tissues, with three to 340 individuals contributing to each library [25]. A pilot study has already been carried out to determine the quality of SNPs detected in the CGP EST collection resulting in the validation of 33 SNPs in two Canadian cod populations [9]. Many of the SNPs developed were identified from sequence data with functional annotation potentially allowing the identification of genes contributing directly to a phenotype.

Putative SNPs identified in this study were subsequently validated for polymorphism across a number of geographically diverse Atlantic cod populations, ranging from Canada to the North-East Atlantic (Iceland, Norway and Ireland). These SNPs were also tested for Mendelian segregation in two families, and used to create a high-density genetic linkage map that can be applied in QTL analysis to facilitate cod broodstock selection.

## Results

### Selection of ESTs and contig assembly

ESTs from the CGP collection were generated using several individuals per library to ensure that a significant proportion of the genetic diversity present in two Atlantic cod populations was captured. These populations originated from Cape Sable (off the southern tip of Nova Scotia) and from Bay Bulls, Newfoundland, and individuals from these collections had been used as parents in breeding programs based in New Brunswick and Newfoundland respectively. Two main methods were used for library generation; normalization of cDNA followed by directional cloning [26,27], with sequencing carried out from either the 3' or the 5' end, and suppressive subtractive hybridisation (SSH) followed by non-directional cloning [28,29]. The sequences chosen for automated SNP discovery originated exclusively from 3'end sequencing of the normalized libraries, as we expected to have fewer splicing events on average, and more opportunity for SNP discovery, in the non-coding 3' untranslated region. The SSH sequences were not used in automated SNP discovery due to the limited genetic diversity within these libraries as each was constructed using fish from a single family [28,29].

The software package Paracel Transcript Assembler (PTA) was used to cluster the EST set. This software groups similar sequences together into clusters, and then attempts to assemble sequences within a cluster [30]. Thus clusters comprise two or more sequences, and can be any combination of 1) one or more contigs 2) two or more singlets or 3) one or more contigs and one or more singlets. Clustering of the 97976 3' ESTs produced 12067 clusters, containing a total of 13448 contigs, and 21746 singlets and singletons, with singlets being defined as single sequences associated with clusters and singletons corresponding to sequences that are unique within this EST set. The contigs were used for SNP discovery. The average number of ESTs that assembled to form a contig ranged from 2 to 83, with an average of 5.66 contributing sequences. A total of 6723 contigs contained 4 or more reads.

### SNP detection

**Figure 1:**
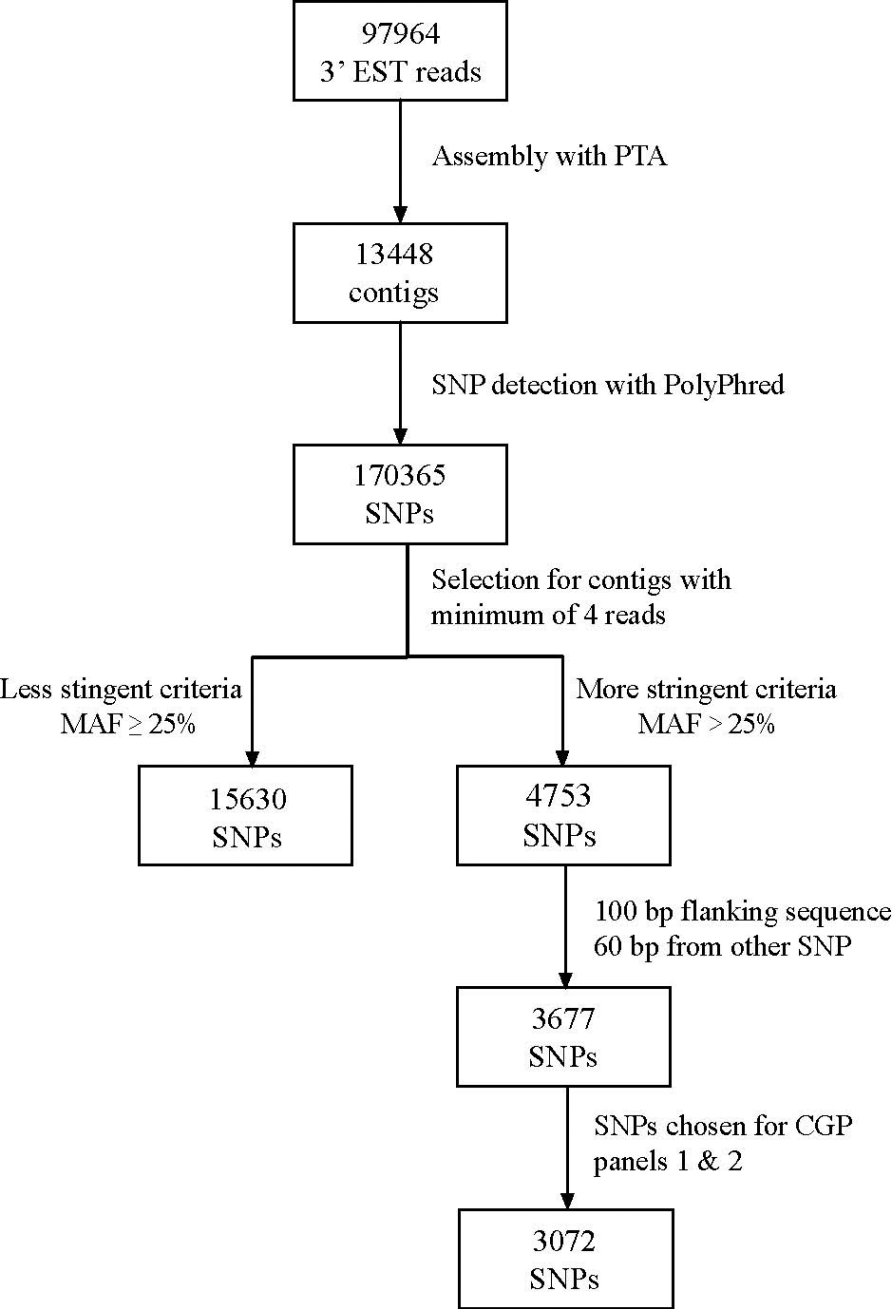
**SNP selection process for CGP panels 1 and 2**. Flowchart showing the selection criteria by which the 3072 SNPs were selected for inclusion on two Illumina Golden Gate panels.

Searching 13448 contigs using an automated pipeline based on PolyPhred yielded a total of 170365 predicted SNPs (Figure 1). To attempt to improve the quality of the SNPs selected for further analysis, selection criteria (minimum 4 read coverage, minor allele frequency (MAF) > 25%) were applied to this initial SNP set to ensure that the minor allele for all SNPs called was represented by at least 2 independent sequences. These criteria were used to reduce selection of false SNPs caused by sequence miscalling or polymerase errors, and to favour the selection of SNPs occurring frequently within the populations under study. Applying our initial selection criteria to all PolyPhred SNPs yielded 4753 SNPs identified from a total of 2723 contigs. The average frequency for this set of predicted good quality SNPs within the contigs used for selection was one per 516 bp.

### Validation of putative SNPs on panel

Out of the pool of 4753 predicted good quality SNPs, 3677 SNPs satisfied the criteria for the Illumina Golden Gate platform in that they appeared to be bi-allelic, with 100 bp of flanking sequence and more than 60 bp from a selected neighbouring SNP (Figure 1), and these SNPs were scored for primer design. Two Golden Gate panels (CGP Panel 1 and CGP Panel 2), each comprising 1536 SNPs (3072 SNPs total), were created from the best-scoring SNPs and these were tested against a large number of Atlantic cod sampled from a number of sites (multiple populations from Canada, and single collections from Iceland, Ireland and Norway). Parents and progeny from two reference families selected from the CGP breeding program in New Brunswick were also genotyped to test for non-Mendelian segregation and for the creation of a genetic linkage map (Table 1).

**Table 1:** Samples genotyped for this study

Description	Breeding program	No. of samples	Purpose
Cape Sable, Canada	NB YC1	23	Population analysis
Bay Bulls, Canada	NL YC2	23	Population analysis
Georges Bank Canada	NB YC2	23	Population analysis
Smith Sound, Canada	NL YC3	23	Population analysis
Akureyri, Iceland		26	Population analysis
Barents Sea, Norway		26	Population analysis
Galway Bay, Ireland		15	Population analysis
Family 33		2 parents, 91 progeny	Segregation analysis Linkage mapping
Family 87		2 parents, 91 progeny	Segregation analysis Linkage mapping

**Figure 2:**
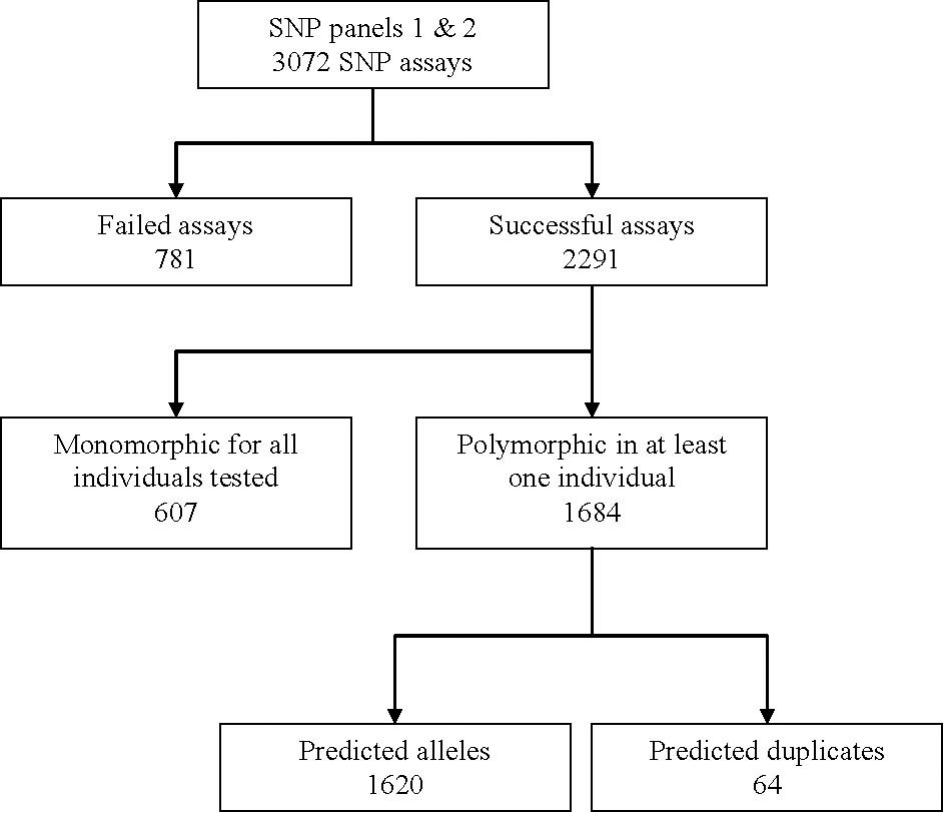
**SNP validation from CGP panels 1 and 2**. Flowchart showing the output resulting from testing of the 3072 selected SNPs. The 1620 "predicted alleles" correspond to the set of validated SNPs.

**Figure 3:**
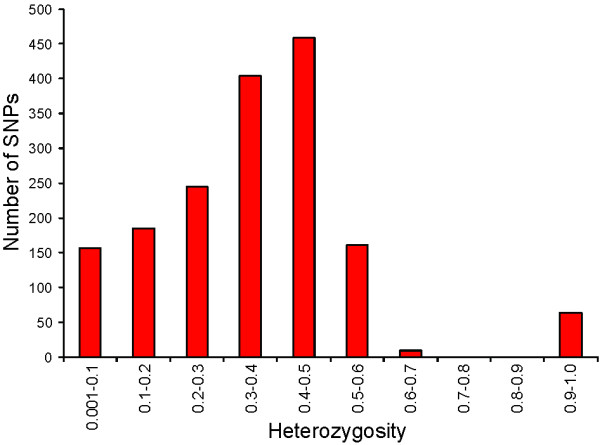
**Observed heterozygosity for polymorphic SNPs in four Canadian populations**. SNPs were grouped into categories based on their values for observed heterozygosity averaged across four Canadian populations. All polymorphic SNPs were analysed, including those with high values for observed heterozygosity (predicted duplicates). The number of SNPs falling into each category is shown.

**Figure 4:**
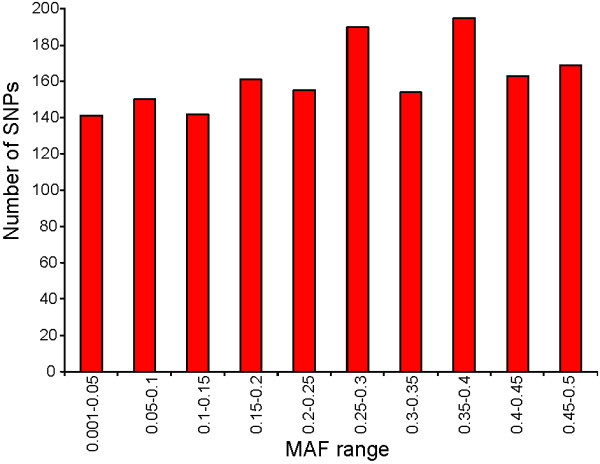
**Minor allele frequency of validated SNPs in four Canadian populations**. SNPs were grouped into categories based on their minor allele frequency (MAF) averaged across four Canadian populations. Only the validated SNPs have been included in this analysis, with the number of SNPs falling into each MAF category shown.

The success rate for SNP assays was 75% for the two panels tested, with a total of 781 assays that failed to give good quality genotypes (Figure 2). From the 2291 successful assays, 607 SNPs (26%) were monomorphic (i.e. only one SNP variant was identified) in all individuals from four Canadian populations that were tested (Table 1), and therefore are either incorrectly identified as SNPs, or are rare SNPs within the populations analysed. The majority of these have a minor allele represented by two sequences in the contigs from which they were identified, the minimum allowed by our selection criteria; only a small number of monomorphic SNPs have more than 2 reads representing the minor allele. In total, 1684 SNP assays identified both SNP variants, with at least one individual tested carrying the predicted minor allele. However, 64 of these SNPs showed a high proportion of heterozygotes in all individuals tested (Figure 3), indicating that they might represent sequence variation between duplicated genes, or members of closely related gene families, rather than different alleles from the same gene; the total number of SNPs predicted as corresponding to bi-allelic loci was 1620 (Figure 2). For the purpose of this study, we define validated SNPs as those having a value for observed heterozygosity greater than zero but lower than 0.9. Therefore this study has identified 1684 polymorphic SNPs, with 1620 of these being validated SNPs as we predict they correspond to a base change at a single position in the genome. The average observed heterozygosity for these validated SNPs was 0.332 (± 0.148) and ranged from 0.01 to 0.69. The mean MAF among the validated SNPs was 0.258 (± 0.141) and ranged from 0.005 to 0.5. The number of SNPs observed for different MAF ranges is shown in Figure 4; the results presented represent the combined results for the four Canadian populations enrolled in the CGP breeding program, with the European populations excluded from this analysis. The number of SNPs in different MAF ranges from 0.05 to 0.5 is relatively consistent for these fish, with 141 validated SNPs having a MAF lower than 0.05, and 169 SNPs with a MAF from 0.45 to 0.5 for example.

SNPs for CGP panel 1 (1536 SNPs) were chosen such that only one SNP per contig was included. The second panel selection consisted of various categories of SNPs chosen based on a prioritized strategy. Initially, SNPs from remaining contigs not represented on the first panel were selected. The panel was then completed by selecting SNPs that are neighbours on the same contig to SNPs that failed, or were monomorphic, on panel 1, and also SNPs that were neighbours to successful SNPs on panel 1 (or a small number of SNPs included on panel 2 which had not yet been tested) but having a different haplotype. A few SNPs were selected manually based on contig annotations, with some of these identified on SSH EST contigs. Thus the final set of validated SNPs (1620) was selected from 1514 contigs, with several contigs having multiple validated SNPs. These can be identified in the SNP set as they have an identical number, but a different suffix, as in cgpGmo-S177a and S177b for example.

SNPs neighbouring failed panel 1 SNPs had slightly lower success rate (69%) when compared to other SNP categories, which ranged from 72 to 79%. Analysis of the polymorphism of successful panel 2 SNPs showed that SNPs neighbouring panel 1 failures have a higher number of polymorphic SNPs (78%) when compared to the "unique SNPs/contig" category (71%). Two categories showing the smallest number of polymorphic loci are the manually picked SNPs (35%) and the neighbours of monomorphic panel 1 SNPs (47%). Some of the manually picked SNPs were selected from SSH libraries which had each been generated from a single family, and thus this subset may contain a greater proportion of SNPs which are rare within the population as a whole.

### Functional annotation of SNPs

The SNPs in the CGP collection are particularly valuable as they are linked to expressed sequences. However, because a large fraction of the 3' sequence in which the SNPs were detected is likely to originate from the 3' UTR of each transcript, most SNPs were expected to fall in non-coding regions. This resulted in a relatively low percentage of sequences for which a function could be inferred based on sequence similarity. Of the 1514 contigs from which at least one validated SNP was selected, 474 (31%) had a significant blast hit (e value ≤ 1 e^-05^) in the NCBI non-redundant (nr) dataset. In total, 514 SNPs (32%) were associated with sequences having significant similarity to an entry in the NCBI nr database (Additional file 1).

After analysis based on sequence homology, a subset of the SNPs identified was found to fall within coding regions; these SNPs were analysed to determine if substitutions encoded by the two allelic variants would result in an amino acid change, i.e, if the substitutions are non-synonymous or synonymous. Only 9% of validated SNPs occur on a known reading frame within coding regions (i.e. they have similarity with a protein sequence present in public databanks). Of these 141 SNPs, 90 (64%) were predicted to generate synonymous substitutions, while 51 (36%) were non-synonymous. For non-synonymous SNPs, the resulting amino acid changes are shown in Additional file 1.

### Population comparison

We present here a description of SNP characteristics in several populations of Atlantic cod (Table 1, Additional file 2). The investigation of cod population structure using these SNPs in these and additional populations will be described in detail elsewhere (Bradbury et al., in preparation). From our analysis, the number of monomorphic loci varied greatly between the Canadian populations and more distant populations such as Ireland and Norway. A large number of loci (1033) are polymorphic in all populations. As anticipated, the greatest number of monomorphic loci from this SNP set is seen in the East Atlantic populations (Iceland, Ireland and Norway). This is likely to be due to ascertainment bias rather than a real underlying difference in variability between West and East Atlantic populations, as SNPs were selected based on their frequent occurrence in Cape Sable and Bay Bulls fish. A number of SNPs (184) have been identified as putative diagnostic SNPs as they have the potential for use in distinguishing between Western and Eastern Atlantic cod populations, being monomorphic in fish tested from the Eastern Atlantic cod populations (Iceland, Ireland and Norway) but polymorphic in Western Atlantic cod populations (Additional file 3).

A few of the polymorphic SNPs were not in Hardy Weinberg equilibrium (HWE) in one or more of the four Canadian populations tested (Additional file 2). We determined that 65 of the polymorphic SNPs significantly deviate from HWE in all four populations (P < 0.05), and the vast majority of these (64) were screened out from the set of validated SNPs as they had values for observed heterozygosity greater than 0.9 and were not included in further analyses. An additional 136 SNPs show significant deviations from HWE in one population only, and this is also true for 19 SNPs in two of the four populations and two SNPs in three of the four populations tested, however these did not exceed the numbers of HWE deviating SNPs expected by chance.

### Mendelian inheritance and informativeness of SNPs for linkage mapping

Segregation patterns of SNPs (Mendelian/non-Mendelian) were tested by genotyping the parents and progeny from 2 CGP families, to ensure that SNPs can be used reliably for linkage analysis. In each case, patterns of segregation were assessed in the 91 progeny genotyped for each family. Different, overlapping sets of SNPs could be assessed for segregation in each of the two families. In family B33 it was possible to examine the inheritance for 858 SNPs, whereas 832 SNPs were informative in family B87 (Additional file 2). The 64 SNPs that were predicted to represent differences between genes (paralogs or members of gene families) were removed prior to analysis of segregation patterns. Additional SNPs also showing non-Mendelian inheritance were screened out prior to generating the linkage groups used for map generation; ten SNPs for B33 and 26 SNPs for B87 were excluded at this stage.

Of the 157 SNPs that deviated from HWE in one, two or three Canadian populations, two failed the test for Mendalian segregation in both families used for mapping (cgpGmo-S1835 and S1962), with a further three SNPs showing departure from Mendelian segregation in family B33 but segregating correctly (within the parameters allowed for Mendelian inheritance) in B87 (cgpGmo-S1219b, S626a and S2232). However, the majority of the SNPs in this second category could be successfully placed on the linkage map.

### Generation of a preliminary genetic linkage map for Atlantic cod

The generation of genomics resources within the CGP program is tightly integrated with family-based selective breeding programs based in New Brunswick and Newfoundland. As part of these programs, individual crosses are generated with known parental contribution, with the progeny from these crosses reared in separate tanks until they reach a suitable size for surgical implantation of a passive integrated transponder tag. Parents and 91 progeny from each of two independent crosses, families B33 and B87, were genotyped using the two Illumina GoldenGate panels described in this study (Table 1) with the aim of generating a SNP-based genetic linkage map.

**Figure 5:**
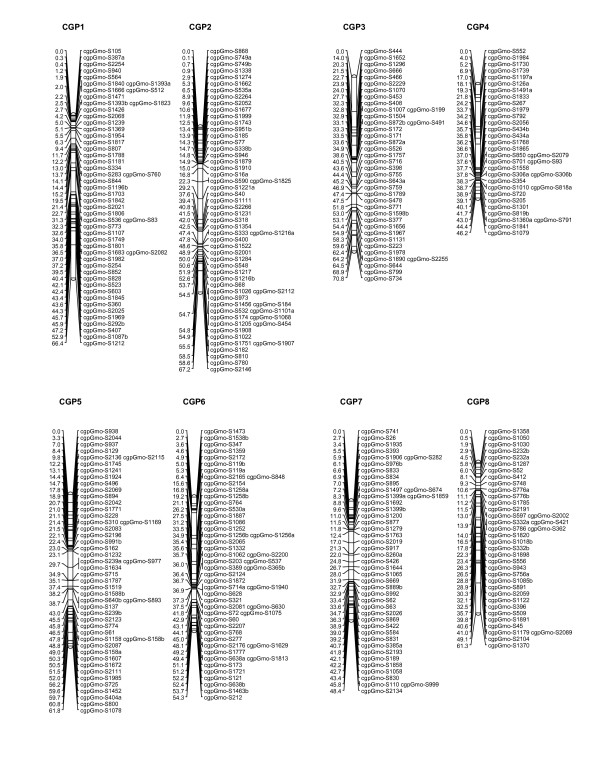
**Genetic linkage map for Atlantic cod (linkage groups 1-8)**. Eight of the 23 major linkage groups are shown. These have been arbitrarily numbered CGP1-8 based on the order generated by JoinMap^®^4, and to distinguish them from the linkage groups generated by Moen and colleagues [24]. Distances in centimorgans are indicated on the left of each linkage group, with SNP identifiers on the right.

**Figure 6:**
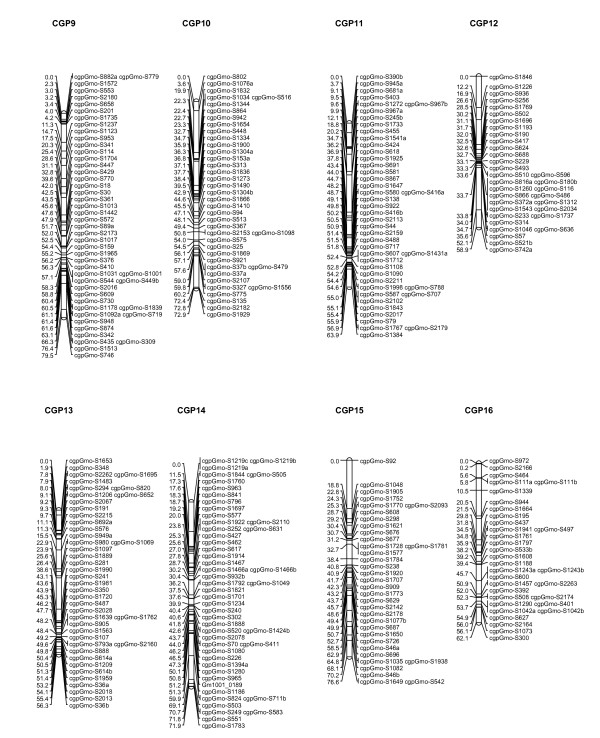
**Genetic linkage map for Atlantic cod (linkage groups 9-16)**. Eight of the 23 major linkage groups are shown. These have been arbitrarily numbered CGP9-16 based on the order generated by JoinMap^®^4, and to distinguish them from the linkage groups generated by Moen and colleagues [24]. Distances in centimorgans are indicated on the left of each linkage group, with SNP identifiers on the right.

**Figure 7:**
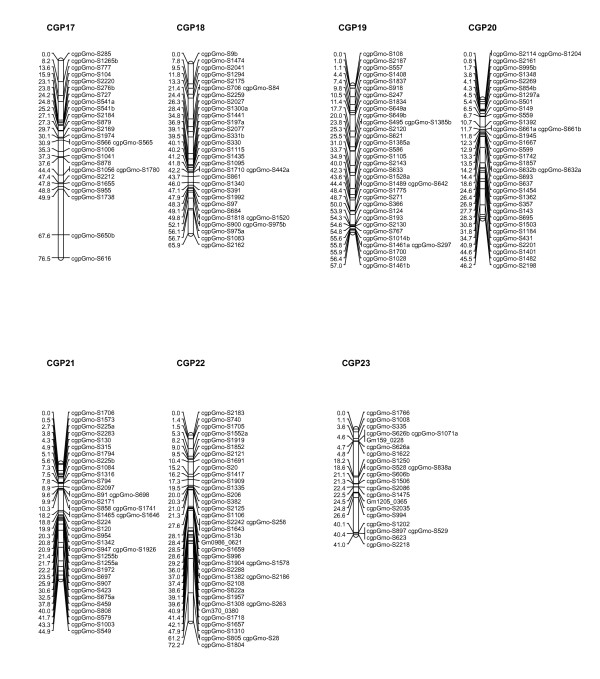
**Genetic linkage map for Atlantic cod (linkage groups 17-23)**. Seven of the 23 major linkage groups are shown. These have been arbitrarily numbered CGP17-23 based on the order generated by JoinMap^®^4, and to distinguish them from the linkage groups generated by Moen and colleagues [24]. Distances in centimorgans are indicated on the left of each linkage group, with SNP identifiers on the right.

After removal of the loci with highly skewed segregation ratios (P < 0.005) described earlier, JoinMap^®^4 [31] was used to generate linkage groups of associated loci for each family independently, and to order loci within linkage groups to create a preliminary map. For both families, 23 major linkage groups were generated using a logarithm (base 10) of odds (LOD) threshold value of 5.0, which is in good agreement with the haploid chromosome number of 23 usually reported for Atlantic cod [32]. A small number of SNPs that failed to be assigned to these 23 linkage groups, as well as a few additional linkage groups generated by JoinMap^®^4 containing 2-3 loci, were not incorporated in further analyses. Marker content of linkage groups, and marker order within those groups, was in good agreement when the maps for the two families were compared. Therefore, the family maps were combined to generate a consensus map using the merge function of JoinMap^®^4. Marker order in the final map was confirmed by analysing the mapping parents for potential double recombinants. The consensus map produced is shown in Figures 5, 6 and 7, and contains 924 loci on 23 linkage groups, ranging from to 41 to 79.5 cM in length, and a total map length of 1421.92 cM. The number of markers per linkage group ranges from 23 to 58, with an average of 40.2.

## Discussion

Our goal was to develop a large collection of SNP markers from contigs produced by the CGP. After screening 13448 contigs generated from 97976 ESTs, we identified 4753 SNPs using the criteria of 4 reads minimum and a MAF > 25%. Assays have been developed for 3072 SNPs using 465 fish, which were genotyped using a Golden Gate assay. The success rate for this set of SNP assays was 75%. The SNPs were assessed for polymorphism by testing against Canadian and European populations and it was determined that 26% of SNPs were monomorphic. A large majority (1610) of the validated polymorphic SNPs described here are novel. However, on analysis, 10 SNPs in our collection were found to be identical to SNPs identified independently in a previous study [23]. These SNPs are listed in Additional file 4, with the original names given by Moen and co-authors [23] together with alternative names used in this study.

The frequency of our set of selected SNPs in Atlantic cod is 1/516 bp, which is similar to the frequency reported in Atlantic salmon of 1/614 bp [16]. It is somewhat lower than the frequency observed in *Oncorhynchus keta *(chum salmon; 1/175bp) or in *Oncorhynchus tshawytscha *(Chinook salmon; 1/301bp) [33]. SNP identification strategy is likely to play a large role in the predicted frequency of SNPs within the genome, but it also might reflect the fact that, in the cases of Atlantic cod and Atlantic salmon, SNPs have been identified from fish originating from a more limited number of populations.

To maximize the detection of real SNPs that were frequently variable in fish enrolled in the CGP breeding programs, stringent criteria were used to reduce the likelihood of selection of false or rare SNPs. By selecting SNPs where the minor allele was represented by at least two reads, we hoped to generate a set of markers that would prove useful for gene mapping and parental assignment. As a consequence, our SNP collection has been selected to prioritise SNPs that are frequently polymorphic in the populations being used for selective breeding in Atlantic Canada, and in related populations from Atlantic Canadian waters. Therefore, our set of SNPs may prove to be less informative for the analysis of populations with different geographical distribution, such as populations originating in North-East Atlantic. The fact that we found 184 SNPs that are polymorphic in Canadian populations but monomorphic in North-East Atlantic populations (Additional file 3) is a clear indication that, due to the ascertainment bias intrinsic within our selection procedure, our collection of SNPs may be less informative in characterizing the genetic structure of European populations. We also anticipate that our SNP collection contains few rare SNPs because of the selection criteria employed. These rare alleles can be useful for the analysis of certain populations since they may prove to be specific to, and thus diagnostic for, these populations.

The SNPs developed in the present study add significantly to the total number of validated SNPs for Atlantic cod. In a previous study, Moen and colleagues identified and validated 318 SNPs [23], however only 10 SNPs were common between the two studies (Additional file 4). The SNPs described in both analyses have been detected from EST assemblies and thus are associated with transcripts. One third of the SNPs were detected on annotated sequences in our analysis as the ESTs on which they were detected have a high proportion of non-coding sequence, whereas in the Norwegian study 87% of the SNPs had a significant BLAST hit. Validation success was similar in both studies, with the percentage of failed assays at 29% for Moen et al. [23] and 25% for our study. The number of polymorphic SNPs as a percentage of all putative SNPs tested was found to be 54% by Moen et al. [23] and 55% in our study (53% for validated SNPs). The number of monomorphic loci was slightly higher in our study than found by Moen et al. [23]. The majority (91%) of predicted SNPs that were found to be monomorphic in the present study have their minor allele represented by 2 reads only. We anticipate that these fall into two categories; 1) SNPs that are rare within the populations tested, and therefore polymorphism at these loci exists but was not observed in the sample set tested, and 2) incorrect SNP predictions. This emphasizes the need for stringent selection criteria and also that validation of SNPs is a necessary step to establish the accuracy of markers.

The libraries from which the sequences used in the assembly were generated, and thus from which SNPs were identified, were created using tissue from fish originating from collections from Nova Scotia (Cape Sable) and Newfoundland (Bay Bulls), Canada. By testing these SNPs against more eastern populations such as Ireland, Iceland and Norway, we have shown that they are also informative as markers across more geographic distant populations. Some SNPs (184) were found to be polymorphic only in all Canadian populations, and therefore have the potential for use as traceability markers.

By genotyping two reference families, SNPs were checked for Mendelian segregation. The 64 SNPs that were removed from the validated set showed a significant departure from Mendelian segregation; they are more likely to occur on paralogous genes than to represent alleles since both parents and progeny were heterozygous. This is not uncommon when identifying SNPs in fish. In most studies around 2-4% of validated SNPs are assumed to be duplication SNPs [23] except for salmon where 14% of SNPs were scored as heterozygotes in all individuals tested [16]. However, in addition to the set of SNPs predicted to occur on duplicated genome segments, several additional SNPs show non-Mendelian segregation patterns in the two families tested. Also, four SNPs, two in family B33 and two in B87, appear to be duplicates in one family, but segregate in the other family, which could be indicative of either selective forces acting differently upon those families or, more likely, complex patterns of gene duplication and divergence.

Most of the SNPs described here are predicted to fall within non-coding sequence. This is expected in our dataset as all of the ESTs used in SNP identification were sequenced from the 3' direction, and thus the majority of each sequence is likely to represent the 3'untranslated region. Nevertheless, a minority of the SNPs identified here are predicted to occur in coding regions. The remaining SNPs are either in non-coding sequence, or on contigs with no significant sequence similarity. For the SNPs found in coding regions, only a subset of the polymorphism will result in a variation in the amino acid sequence of the encoded protein (i.e. the non-synonymous substitutions). SNP studies have reported a higher number of synonymous SNPs (sSNPs) when compared to non-synonymous SNPs (nsSNPs); the variation at non-synonymous sites has the potential to be associated with deleterious mutations. A higher number of sSNPs is usually observed, and this is likely to be the result of evolutionary constraints preferentially eliminating variation at non-synonymous sites. For example, 80% of SNPs identified in coding regions in chicken [34] are synonymous compared to 71% for *Schistosoma mansoni *[35], 68% for *Anopheles funestus *[36], 60% for zebrafish [37], and 55% for rat [38]. An even higher frequency of sSNPs has been detected in *Salmo salar *(82%) [16]. We found that the frequency of sSNP in Atlantic cod (64%) is intermediate to that reported for other species.

A preliminary linkage map has been constructed using the SNPs presented here. This map has been generated using the cross-pollination (CP) parameter set of JoinMap4^® ^[31], which is applicable to crosses generated from wild individuals taken from an outbred population, and has also been used to generate maps from a small number of crosses in other species [39,40]. Independent maps were created from the two families B33 and B87, which gave the same number of major linkage groups (23) and a similar overall marker order. Maps generated from these two families were merged to give the consensus map shown in Figures 5, 6 and 7. Preliminary analysis of additional families on a second-generation SNP panel (results not shown) gives additional support to this consensus map.

Prior to this analysis, Moen and colleagues [24] described an integrated genetic linkage map for Atlantic cod. This map was constructed using both SNPs and microsatellites, and comprises 25 linkage groups with 207 mapped markers in total. Unfortunately, it is not currently possible to cross-reference the linkage groups presented in this report with the groups generated in the present study, as too few markers are common between the different maps. We are currently in the process of map refinement, with the aim of adding further families and incorporating a large number of the published microsatellite markers available for Atlantic cod, which should allow integration of these two mapping efforts.

We can generate separate male and female maps for most of the genome of Atlantic cod using the two families genotyped on the two SNP panels described here. The majority of the linkage groups in the consensus map could be identified in sex-specific maps, however these maps are less dense and, due to their bi-allelic nature, only a few informative SNPs are common between maps created with a single individual, making the merging of maps problematic. However, although there appears to be a significant difference in the recombination rates between male and female Atlantic cod [24], this has not prevented construction of an integrated map both here and in the previous study [24].

This large collection of SNPs for Atlantic cod should prove of great utility for both the aquaculture industry, and for the management of wild fisheries. As improved automated genotyping systems have been developed, SNPs have become important markers for commercial diagnostics and parental genotyping applications. Due to lower individual information content, a higher number of SNPs is required for parental assignment [41] when compared to the microsatellite marker approach that is the current industry standard. In pigs, comparable parental exclusion probabilities have been achieved when using a panel of 60 SNPs or a 10 microsatellite marker panel, but the SNP panel was more sensitive for individual identification [42]. In cattle, panels of 32 and 37 highly informative SNPs were powerful enough to distinguish progeny from multibreed composite populations [41,43]. To develop a powerful SNP panel for cod parental assignment, SNPs selected for inclusion in that panel should have a high MAF within the families subject to analysis [41]. In total, 332 SNP markers developed by the CGP have a MAF higher than 0.4 (Figure 4). We have selected a subset of these SNPs to develop a SNP panel for use in relatedness analysis, parental assignment and product traceability applications within the cod aquaculture industry. It should also be possible to apply this large marker set to increase the resolution of population structuring within wild populations of Atlantic cod, and to better monitor the genetic diversity within populations that are being actively fished.

The SNP collection presented here has been completed in parallel with the development of a 20000 feature, oligonucleotide microarray. In the CGP microarray, 1391 features have a validated SNP marker, which will lead to the association of features showing interesting transcriptional responses with QTL intervals, potentially providing useful tools for MAS. For example, cgpGmo-S1123 is located in the sequence coding for 3-oxo-5-beta-steroid 4-dehydrogenase (AKR1D1). This gene belongs to the Aldo-keto reductase family 1, member D1 and catalyzes the reduction of progesterone, androstenedione, 17-alpha-hydroxyprogesterone and testosterone to 5-beta-reduced metabolites, as well as playing a role in bile acid biosynthesis [44]. This gene is of great interest for its role in sexual maturation, and MAS for selected variants can potentially be developed using the associated SNP.

The SNPs described here have been derived from ESTs, and thus can provide anchor points for more extensive comparative genomic analyses. A second generation SNP array (1536 SNPs) has now been created by selecting SNPs from these two initial panels (CGP panels 1 and 2) to produce a third Golden Gate panel comprising validated, polymorphic SNPs (CGP panel 3). This is currently being used for QTL detection.

## Conclusions

We present an extensive resource of SNP markers for Atlantic cod, *Gadus morhua*. The SNPs have been validated across a panel comprising several populations of wild cod, and using two family crosses. This large collection of SNPs will be valuable for developing diagnostic assays to distinguish between cod populations, as well as producing tools useful for the aquaculture industry. A dense genetic linkage map has been constructed using these SNP markers and will provide a valuable resource for QTL discovery and MAS.

## Methods

### EST libraries, clustering, contig assembly and annotation

The creation and the sequencing of the Atlantic Cod Genomics and Broodstock Development Project (CGP) EST libraries have been described in detail elsewhere [25]. Libraries were derived from fish taken from populations originating from Eastern Canada (Cape Sable, NS and Bay Bulls, NL) that were also enrolled in two breeding programs located at the Huntsman Marine Science Centre, NB and the Joe Brown Aquaculture Research Building, Ocean Sciences Centre, Memorial University, NL, respectively. Samples for library generation were taken from multiple tissues from adult fish from either 1) the same collection as fish enrolled in the breeding programs, 2) from parents of family fish, 3) from F1 juveniles produced by the breeding programs. ESTs used for SNP identification were generated from normalized cDNA libraries that were directionally cloned into the vector pDNA-LIB (Clontech, Mountain View, CA) and were sequenced from the 3' end of the transcript. The clustering process by which contigs were generated for SNP prediction has been described in detail previously [9].

All EST sequences used in this study have been deposited in GenBank [25]. Annotated sequences are also available from the project database at http://ri.imb.nrc.ca/codgene.

### SNP detection

Automatic SNP detection has been described in detail previously [9]. Contigs generated from the clustering process were searched for SNPs using PolyPhred [45]. Output files generated by PolyPhred were parsed using a custom Perl script to extract information regarding location of putative SNPs, read coverage at the SNP position, and the proportion of contributing sequences with each sequence variant. SNPs detected by PolyPhred were filtered for quality by selecting SNPs with a minimum of 4 read coverage at the SNP position and a MAF greater than 25%. This ensures that at least two individual reads must have the minor allele for a contig containing 4 reads. These criteria were designed to maximise the selection of frequently polymorphic SNPs, and to reduce the risk of selecting false SNPs due to sequencing errors. Further refinement of SNP selection was performed to accommodate the requirements of the Illumina GoldenGate SNP genotyping platform. SNPs with less than 100 bp of flanking sequence on either side, or within 60 bp of another selected SNP, were removed from consideration (Figure 1). In addition, only bi-allellic SNPs were selected for GoldenGate assay genotyping.

### Validation of putative SNPs on panel

In total, 5 × 96 well plates of selected DNA samples were genotyped using the two Illumina Golden Gate panels. Two plates consisted of two references families, B33 and B87 with two parents and 91 progeny. The three remaining plates consisted of wild cod populations. In total, seven populations of Atlantic cod were genotyped for this study, with an average of 23 fish genotyped per population. The geographic location of collections covers the North Atlantic with a more detailed sampling for Atlantic Canadian populations. DNA extraction methods have been described previously [46]. In summary, fin clips or muscle tissue samples were taken and placed in 95% ethanol. DNA was extracted using the Qiagen DNAeasy 96 extraction kit (Qiagen, Mississauga, ON). The kit protocol utilizes a buffer containing proteinase K to lyse the tissue. The lysate was loaded onto a plate where the DNA binds to a silica membrane in the presence of chaotropic salt. Proteins and other contaminants were washed from the bound DNA using wash buffers and centrifugation. DNA was then eluted in water.

High-throughput genotyping was performed at the McGill/Genome Quebec Innovation Centre using the GoldenGate assay.

### SNP annotation

For sequences where SNPs were detected, the consensus sequence for each contig was compared to the NCBI nr database using BLASTX [47], with a value of 1 × e^-05 ^used as the cutoff to determine significance. All SNPs that were determined to be polymorphic after testing have been deposited in the GenBank SNP database under accession numbers ss131570222 to ss131571915 (Additional file 2). Sequences, and their associated annotation using both BLASTX and AutoFACT [48], can also be accessed via the CGP database http://ri.imb.nrc.ca/codgene.

### Identification of synonymous and non-synonymous SNPs

The procedure for determining a SNP as synonymous or non-synonymous is outlined in Hubert et al., 2009 [9]. Briefly, each contig consensus was compared against the NCBI protein database using BLASTX to establish a reading frame in which to assess synonymous or non-synonymous status. For those SNPs within regions of similarity, the consensus sequence was translated for each SNP allele and the resulting amino acid sequences were then compared to determine whether the SNP was synonymous or non-synonymous.

### Analysis of Atlantic cod populations

Loci deviating from Hardy-Weinberg equilibrium (HWE) were identified in each of four Canadian populations of Atlantic cod. This was assessed separately in each population using Hardy-Weinberg exact tests calculated using GenePop v4.0 [49]. Loci with calculated P-values less than 0.05 were considered to deviate from HWE in that population (Additional file 2).

### Genetic linkage map construction

The genetic linkage map was constructed using JoinMap^®^4. Genotypes for progeny generated through the Illumina GoldenGate platform were converted to CP codes based on parental genotypes. Each cross was examined separately, with segregation ratios analysed for all loci, and those which showed abnormal segregation as determined using a chi-square goodness of fit test were removed (P < 0.005). Markers were then associated within linkage groups using the group function of JoinMap^®^4, using a LOD cut-off value of 5.0 or greater. Marker orders within linkage groups were determined and map distances calculated using Haldane's mapping function. Maps generated independently for the two families were compared, and a 1:1 correspondence between linkage groups confirmed. The corresponding groups from the two families were combined using the JoinMap^®^4 merge function, and a consensus map generated. This map was generated from the first round of JoinMap^®^4, which integrates markers that score highly for goodness-of-fit; no attempt was made to force additional loci with a reduced goodness-of-fit into the map.

The marker order from the merged map generated was used to check maps generated individually for the four contributing parents to identify suspicious double recombinants that would indicate potential errors in the marker order. This was performed using the "Create Maternal and Paternal Population Nodes" function of JoinMap^®^4, followed by the generation of linkage group-specific maps for markers informative in each parent. The marker order in the merged map was retained in the parental maps by inputting this as a fixed order to direct map generation, with progeny then analysed to detect double recombination events.

## Authors' contributions

SH performed the initial selection of SNPs for inclusion in this study, analysed the data from the SNP panels and co-wrote the paper. BH was involved in the initial selection of SNPs for inclusion in this study, wrote custom Perl scripts which were used for SNP selection and analysis and was involved in drafting the paper. TB assisted with the analysis of cod populations and with generation of the genetic linkage map and helped with the drafting of the final versions of the paper. SB was responsible for the conceptualization, design and implementation of the SNP identification and analysis program, generated the genetic linkage map, and co-wrote the paper. All authors read and approved the final manuscript.

## Supplementary Material

Additional file 1 **Automated annotation of SNP-containing contigs and amino acid substitutions for non-synonymous SNPs**. This includes best hit in the NCBI nr database together with the bit score and e-value, and alternative amino acids for any SNPs resulting in non-synonymous substitutions. Click here for file

Additional file 2 **Properties of selected SNPs for which a functional assay was developed**. This includes SNP accession numbers, observed and expected heterozygosity, minor allele frequency, tests for departure from HWE and Mendelian segregation. Click here for file

Additional file 3 **Atlantic cod SNPs polymorphic in Atlantic Canada but monomorphic in the eastern Atlantic**. List of SNPs that were monomorphic in the eastern Atlantic (Iceland, Ireland and Norway) but polymorphic in Atlantic Canadian populations of cod. Click here for file

Additional file 4 **Previously published SNPs**. Names of previously published SNPs together with any alternative names for these SNPs that were used in this study. Click here for file
